# Cortical White and Grey Matter Volume Differences Associated with Plasma Cytokine and Chemokine Levels in PLWH in Cape Town

**DOI:** 10.3390/ijms262412000

**Published:** 2025-12-13

**Authors:** Vurayai Ruhanya, Susan Engelbrecht, Monray E. Williams, Robert H. Paul, Justen Manasa, George Nyandoro, John A. Joska, Soraya Seedat, Richard Helmuth Glashoff

**Affiliations:** 1 Division of Medical Virology, Department of Pathology, Faculty of Medicine and Health Sciences, Stellenbosch University, Cape Town 8000, South Africa; susanen@sun.ac.za; 2 Medical Microbiology Unit, Department of Laboratory Diagnostic and Investigative Science, Faculty of Medicine and Health Sciences, University of Zimbabwe, Avondale, Harare PO Box A178, Zimbabwe; jmanasa@medsch.uz.ac.zw (J.M.); georgenyandoro@yahoo.com (G.N.); 3 Biomedical and Molecular Metabolism Research (BioMMet), North-West University, Potchefstroom 2531, South Africa; monray.williams@nwu.ac.za; 4 Department of Psychology and Behavioral Neuroscience, University of Missouri-St Louis, 1 University Blvd, St. Louis, MO 63121, USA; robert.paul@mimh.edu; 5 MRC Unit of Anxiety & Stress Disorders, Department of Psychiatry & Mental Health, University of Cape Town, Cape Town 8000, South Africa; john.joska@uct.ac.za; 6 MRC Unit of Anxiety & Stress Disorders, Department of Psychiatry, University of Stellenbosch, Cape Town 8000, South Africa; sseedat@sun.ac.za; 7 Division of Medical Microbiology, Stellenbosch University, Cape Town 8000, South Africa; rglas@sun.ac.za; 8 National Health Laboratory Service (NHLS), Tygerberg Business Unit, Cape Town 8000, South Africa

**Keywords:** Luminex, cytokines, chemokines, inflammation, MRI, cognitive impairment

## Abstract

HIV infection is accompanied by production of pro-inflammatory cytokines, which are regarded as critical in neuronal damage, leading to brain dysfunction. To develop diagnostic tools and therapeutic interventions, we need to measure CNS response to immune activation, hence the need to identify specific cytokine biomarkers that are associated with brain damage in HIV infection. This cross-sectional retrospective study applied Magnetic Resonance Imaging (MRI) for brain volumetric measurements and high-throughput Luminex-based immunoassays to quantify plasma cytokine and chemokine concentrations. We then used generalized linear models and Partial Least Square Regression models to evaluate the association between brain volume and plasma cytokines in predominantly treatment-naïve participants with HIV. After adjusting for clinical and demographic variables, we observed that higher MCP-1 (*p* = 0.013) and RANTES (*p* = 0.002) remained significantly associated with lower cortical white matter volume, whereas the anti-inflammatory cytokine IL-9 (*p* = 0.025) and the growth factors PDGFBB (*p* = 0.012) and VEGF (*p* = 0.001) were associated with higher cortical white matter volume. Only IL-6 (*p* = 0.010) was significantly associated with lower subcortical grey matter volume. Higher concentrations of five pro-inflammatory cytokines, IL-6 (*p* = 0.0001), IL-8 (*p* = 0.018), GCSF (*p* = 0.004), MCP-1 (*p* = 0.004), and RANTES (*p* = 0.015), were associated with lower total grey matter volume. Associations of pro-inflammatory cytokines with lower brain volume could imply a link to mechanisms of HIV-associated brain damage, which may lead to neurocognitive impairment. Therefore, the use of highly sensitive neuroimaging and high-throughput immunoassays in HIV-associated brain disorders has potential applications in clinical assessments and therapeutic monitoring.

## 1. Introduction

Despite the immense contribution of combination antiretroviral therapy (cART) to reducing morbidity in people infected with HIV, people living with HIV (PLWH) continue to develop a spectrum of cognitive, motor, and psychological manifestations, clinically referred to as HIV-associated neurocognitive disorders (HANDs). This condition affects more than 50% of PLWH [1,2]. The pathology of HANDs is not completely understood, but it is thought to be a complex interaction of virus, inflammation, and host immune response [3]. Pathological evidence of brain invasion by HIV includes neural loss, dendritic damage, astrogliosis, microgliosis, and nucleated giant cell formation [4]. HIV is thought to penetrate the central nervous system (CNS) through the trafficking of cell-free virus by transcytosis across the blood–brain barrier (BBB) and through the disrupted BBB caused by HIV infection. It is thought that BBB disruption is enhanced by a peripheral pro-inflammatory environment, which is induced by HIV [5]. Transport of cytokines across the BBB has been demonstrated for IL-1α, IL-1β, IL-6, and tumour necrosis alpha (TNF-α) [6]. This passage of blood-borne cytokines to the brain can potentially affect brain integrity and function, with cytokines altering the integrity of the BBB and compromising the ability to regulate the trafficking of immune cells [7,8]. Therefore, peripheral inflammatory cytokines may be putative mediators of neuroinflammation in HIV infection. A link between peripheral inflammation and increased cytokine production in the CNS has been demonstrated in HIV infection [9]. It has been suggested that the major impact of peripheral inflammatory cytokines on the CNS may possibly be through cytokine-mediated production of prostaglandins in the brain endothelium [10]. 

Recently, Magnetic Resonance Imaging (MRI) techniques have shown greater potential in understanding the mechanisms underlying HIV-associated neurocognitive impairment [11]. MRI technologies which are non-invasive can generate objective brain measurements that are able to determine the extent of neurodamage associated with HIV infection. Fully automated MRI technologies have been developed for segmentation of the brain [12,13] in which specific brain regions of interest are assessed for abnormal structural changes in people with HIV [14]. The advantages of segmentation-derived brain volumetric measures are non-invasiveness and high-throughput in investigating brain changes in HIV infection [15,16]. It has been observed that volumetric changes correlate with neuropsychological and clinical abnormalities in HIV-associated cognitive impairment [17]. Grey and white matter atrophy has also been reported in PLWH [18,19,20,21]. Primary brain regions that have been shown to be affected by neuroinflammation are the hippocampus and the entorhinal and temporal cortices [22]. The structural imaging of the brain of PLWH has shown impact on grey matter structures and subcortical regions, as well as cerebral atrophy with ventricular enlargement [23,24,25]. This study applies volumetric neuroimaging and high-throughput Luminex-based immunoassays to examine the relationship between specific brain regions and plasma cytokine levels to assess the inter-relatedness of brain changes in HIV and concomitant peripheral immune activation in predominantly treatment-naïve PLWH. We hypothesized that higher levels of pro-inflammatory cytokines are associated with lower volume of specific brain regions.

## 2. Results

### 2.1. Clinical and Demographic Characteristics

In this study we enrolled 156 participants: 74 % (n = 115) females and 26% (n = 41) males. Approximately 37% (n = 58) of participants had neurological impairment. In the same cohort, 83% were treatment-naïve, whilst 17% were on ART at the time of recruitment, clinical assessment, and sample collection. Participants’ average age was 31.7 ± 5.3 years, and they had high viral load, with an average of 124,750 ± 21,515 RNA Copies/mL, and a low CD4+T-cell count, on average 241.7 ± 174.0 cells/μL; see Table 1. Pairwise correlations to evaluate the relationship between clinical parameters and brain volume showed no significant correlations between brain volume and clinical parameters; see Table 2.

### 2.2. White Matter Volume as a Function of Plasma Cytokine Levels

**Figure 1:**
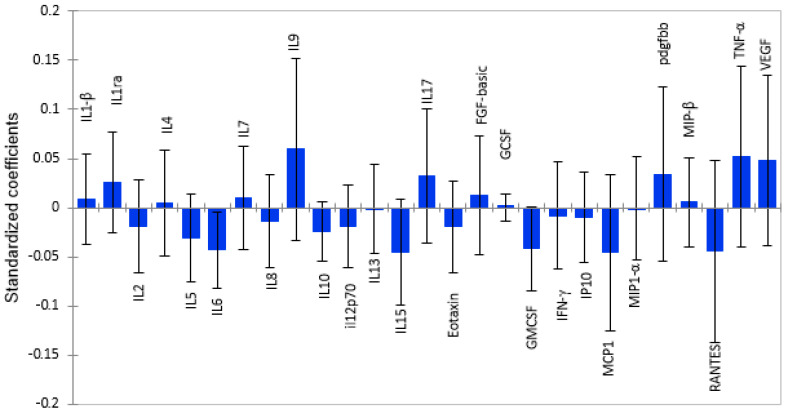
Graphical representation of the magnitude and direction of plasma cytokine associations with cortical white matter volume using PLS-R standardized coefficients with 95% confidence intervals. Cytokines with standardized coefficients above zero show positive associations, and those below standardized coefficients show inverse relationships.

Using Partial Least Squares Regression (PLS-R) modelling, we identified twelve pro-inflammatory cytokines/chemokines that were associated with lower cortical white matter volume, as shown by the standardized coefficients in Figure 1. Among these pro-inflammatory cytokines, MCP-1, RANTES, GM-CSF, and IL-6 had the strongest negative correlation with cortical white matter, as shown by the magnitude and direction of the bars. Anti-inflammatory cytokines and growth factors were associated with higher brain volume, with IL-9 having the strongest positive associations with cortical white matter volume. However, only MIP-1β (β = 0.005; *p* = 0.029) had a significant association. The rest of the cytokines did not show significant associations with brain volume, as shown in Table S1

### 2.3. Subcortical Grey Matter Volume as a Function of Cytokine Levels

**Figure 2:**
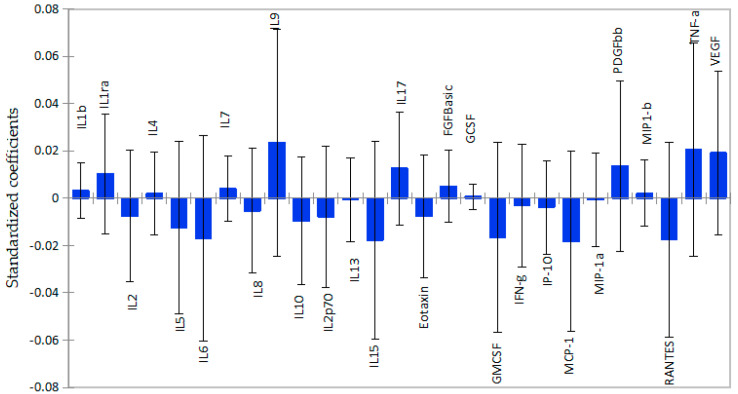
Graphical representation of the magnitude and direction of plasma cytokines’ impact on subcortical grey matter volume using PLS-R standardized coefficients with 95% confidence intervals. Inflammatory cytokines were associated with lower subcortical grey matter volume, as shown by standardized coefficients below zero. Anti-inflammatory cytokines and growth factors with coefficients above zero show positive association.

The associations between plasma cytokines and subcortical grey matter volume followed the same pattern as the ones observed in cortical white matter, although the regression coefficients were smaller for subcortical grey matter than for cortical white matter, as shown in Figure 2. From a panel of 27 cytokines, only 5 pro-inflammatory cytokines, IL-2 (β = −0.008; *p* = 0.012), IL-6 (β = −0.0.017; *p* = 0.002), IL-8(β = −0.005; *p* = 0.018), IL-13 (β = −0.001; *p* = 0.05), and GM-CSF (β = −0.016; *p* = 0.03), were significantly associated with lower volume, as shown in Table S2. In contrast, the cytokines IL-1β (β = 0.003; *p* = 0.046), IL-7(β = 0.004; *p* = 0.014), and growth factor FGF-basic (β = 0.005; *p* = 0.039) were significantly associated with higher volume of subcortical grey matter. Although IL-1ra, IL-9, VEGF, and TNF-α showed relatively strong positive associations with subcortical grey matter, the associations were not significant. Interestingly, IL-10, an anti-inflammatory cytokine, correlated with lower subcortical grey matter volume, but the association was not significant.

### 2.4. Total Grey Matter Volume as a Function of Plasma Cytokines

**Figure 3:**
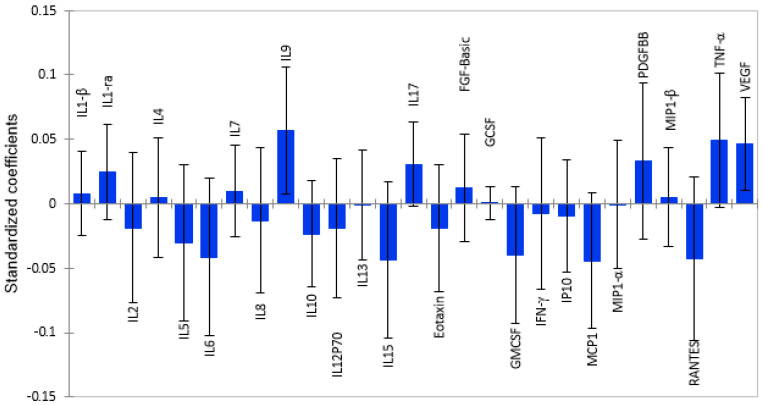
Graphical representation of the magnitude and direction of plasma cytokines’ impact on total grey matter volume using Partial Least Squares Regression (PLS-R) standardized coefficients. Pro-inflammatory cytokines with correlation coefficients below zero show associations with lower brain volume, while anti-inflammatory cytokines show positive associations.

Thirteen pro-inflammatory cytokines and chemokines, namely, RANTES, MCP-1, IP-10, IFN-γ, Eotaxin, GM-CSF, IL-2, IL-5, IL-6, IL-8, IL-12p70, IL-13, and IL-15, were associated with lower grey matter volume, as shown in Figure 3. However, only four cytokines, IL-6 (β = −0.041; *p* = 0.019), IL-8 (β = −0.013; *p* = 0.023), GM-CSF (β = −0.040; *p* = 0.054), and Eotaxin (β = 0.019; *p* = 0.035), were statistically significant, as shown in Table S3**.** Four anti-inflammatory cytokines and three growth factors had positive associations with total grey matter volume. However, only PDGF-BB (β = 0.033; *p* = 0.053) showed a tendency towards a significant association with total grey matter volume. Similar to its relationship with subcortical grey matter, Interleukin 10 was associated with lower, total grey matter volume, but it was not statistically significant.

After adjusting for age, sex, plasma viral load, and CD4+T absolute count, only four anti-inflammatory cytokines and two growth factors remained significantly associated with higher cortical white matter volume. The pro-inflammatory cytokines MCP-1 and RANTES were associated with lower cortical white matter volume after adjusting for the same clinical and demographic parameters. IL-6 remained significantly associated with lower subcortical grey matter volume, while IL-1α was associated with a higher volume of the same brain region. Nine pro-inflammatory cytokines showed significant inverse relationships with total grey matter volume, as shown in Table 3. 

## 3. Discussion

Brain volume loss is widespread in neurological disease; hence, it can be a useful measure of CNS damage and a marker of clinical disease progression. In particular, cortical brain volume loss has been used as a marker to detect overall structural changes during the disease process. We examined the relationship between plasma cytokine concentration and cortical white matter and grey matter volume in pre-treated HIV infection. Our findings show that RANTES was the strongest predictor of low cortical white matter and cortical grey matter volume, followed by IL-2, Eotaxin, IL-8, and MCP-1 IL-6 and GM-CSF. All of them are pro-inflammatory cytokines and chemokines. Brain volume reduction is thought to be influenced by a pro-inflammatory environment, which is partly caused by blood-derived activated monocytes/macrophages and their associated pro-inflammatory cytokines [26]. The structural brain imaging of patients suspected of HIV-associated neurocognitive impairment has revealed a profound impact on grey matter, subcortical regions, and cerebral atrophy [27].

PLWH have elevated levels of systemic inflammatory markers, due to persistent viral replication, release of progeny virus, and viral proteins from infected cells, with clear effects on white matter microstructure [28]. Brain structural studies in HIV infection have shown decreased cortical white matter volume [29] and subcortical grey matter with executive deficits [30]. The results of this study clearly demonstrate an inverse association between brain volume and the peripheral pro-inflammation environment, implying that there is a link between systemic levels and HIV-associated brain volume alterations. Chemokine-mediated inflammation is known to cause gliosis and dendritic damage in HIV [31], and cortical thinning on MRI was shown to be a sensitive index of declining neurological and immune function in patients with AIDS [32]. Even mild dendritic loss may lead to behavioural alterations in HIV-associated minor cognitive motor disorder. Our previous studies have shown that plasma pro-inflammatory cytokines/cytokines, particularly RANTES, IP-10, and IL-2, were strong correlates of HIV- associated cognitive impairment and have shown significant clinical validity as candidates for HAND diagnosis [33].

We have demonstrated that lower cortical white matter and total grey matter volume in patients with HIV were associated with higher plasma RANTES concentration, but the magnitude differed between the brain centres. HIV-related neurocognitive impairment is associated with cerebral atrophy [33]. Therefore, neuroimaging and measures of systemic inflammation such as RANTES have a potential for further investigations as diagnostic alternatives for HIV-related CNS injury. In fact, higher levels of RANTES have been observed in brain lesions in patients with HIV and are expressed in several inflammatory diseases of the CNS [34].

Monocyte chemoattractant protein-1 (MCP-1/CCL2) showed a negative correlation with both cortical white matter and total grey matter volume. The link between MCP-1 and neurodamage was shown in HIV encephalitis (HIVE) and was increased in the brain of patients with AIDS and HIV-associated dementia (HAD) [35]. It has been shown to play a major role in blood–brain barrier disruption, and higher levels in CSF were associated with glial dysfunction [36]. Our study demonstrates an association between plasma MCP-1 and lower brain volume, implying a degree of brain injury in HIV infection, which may lead to cognitive decline. Higher plasma MCP-1 levels have been associated with greater severity and faster cognitive decline in HIV [37]. Therefore, plasma MCP-1 might reflect the risk and progression of HIV-associated neurodamage. Ongoing brain injury may be subclinical for long periods in HIV infection, but the use of inflammatory biomarkers like MCP-1 and MRI technologies can quantify the degree of neurological involvement. The advantage of such approach is that MRI is non-invasive, plasma samples for cytokines are easy to access, and this biomarker is easy to measure.

IL-6-mediated inflammation is known to cause higher incidence of gliosis and dendritic damage in patients with neurological diseases such as Parkinson’s disease (PD) and Alzheimer’s disease (AD) [38]. The observed association between elevated plasma IL-6 levels and significantly lower cortical white matter and grey matter volume in HIV infection suggests that related processes in PD and AD pathology are involved. Based on PLS-R, IL-6 had the strongest association with lower cortical white matter volume, followed by total grey matter volume and lastly subcortical grey matter volume. However, only associations of IL-6 with subcortical grey matter and total grey matter were significant. This implies that cortical grey matter and white matter loss may be partly linked to IL-6-mediated inflammation, but the magnitude is different. The volume of cortical grey matter has also been shown to be significantly negatively correlated with IL-6 in schizophrenia [39]. Higher levels of IL-6 in older adults have been cross-sectionally and longitudinally associated with cortical thinning, cognitive impairment, and increased dementia risk [40]. These findings underscore our observations of a link between the cytokine and neurological conditions. However, the consequences of higher IL-6 on neuronal and glial health and the integrity of cortical volume in the context of HANDs need to be further investigated to accumulate sufficient data for use in clinical investigations.

Contrary to the inverse relationship between pro-inflammatory cytokines and brain volume, anti-inflammatory cytokines such as IL-9 and growth factors were significantly associated with higher brain volume. IL-9 is a pleiotropic cytokine produced by a variety of cells [41]. We suggest that the association between higher IL-9 and larger brain volume might be due to its neurotrophic and neuroprotective role. This cytokine might also play an important role in brain tissue regeneration and repair in HIV infection. IL-9-mediated interference with inflammation and tissue repair could be further investigated to elucidate the neuroprotective mechanisms in HIV infection and exploring strategies of IL-9 immunotherapy in HIV-associated neurocognitive impairment.

VEGF, produced by many cells, is both a potent angiogenic factor and mitogen and has been demonstrated to directly stimulate tumour cells to induce apoptosis resistance [42]. We observed that higher levels of this growth factor were associated with higher brain volume. This growth factor has been shown to have neurotrophic effect and enhances survival of neurons in some brain regions, and higher levels have been detected in HIV-associated CNS diseases [43]. Another growth factor related to VEGF, platelet-derived growth factor (PDGF)-BB, was also associated with higher volumes of both cortical white matter and grey matter volume. This growth factor has demonstrated promotion of neuronal proliferation and reversal of neurotoxicity mediated by HIV-1 Tat [44]. This growth factor is also involved in astroglial scar formation, which confines inflammation to the lesion core and protects the neuronal tissue [45]. Although both VEGF and PDGFBB had positive associations with brain volume, the strength of the PDGFBB association was less than that of VEGF. We suggest that the results can guide further clinical studies on the neuroprotective role of both PDGF-BB and VEGF in HIV infection. However, it would be interesting to determine the neuropsychological correlates of plasma VEGF and PDGF-BB to assess how these growth factors impact the neurobehavioural aspects of HIV. Neuropsychological correlates of VEGF would enable us to confirm that the observed links between higher plasma levels of these growth factors and higher cortical white matter and grey matter volume are the result of a biological process.

Although TNF-α was associated with higher volume of all brain areas, i.e., cortical white matter, subcortical grey matter, and total grey matter, the association was only significant in total grey matter. TNF-α is an inflammatory cytokine whose neurotoxic role in HIV-associated neurocognitive disorders is well documented [46,47]. The implications of our findings are contrary to the neurodegenerative role of TNF-α, as it was associated with increased brain volume. Our findings seem to portray a neuroprotective or neurotrophic role of TNF-α in HIV infection. The neuroprotective role of TNF-α has been demonstrated in experimental models [48] and humans with Alzheimer’s and vascular dementia. Detrimental or beneficial effects of cytokines depend on concentration and time of expression, among other factors, and this could determine the final effect on the CNS. We suggest that future studies could perform threshold analysis for direct association studies between cytokines and brain volume in order to determine which levels are protective or neurotoxic. While this study focused mainly on Xhosa-speaking individuals, which enhances internal validity, it limits external generalizability of the findings to other populations and ethnic groups.

This study focused on the correspondence between peripheral cytokines and chemokines on brain integrity among PWH. The results show that differential patterns of peripheral cytokine/chemokine activation are associated with altered brain volume, implying their effect on neural integrity in PLWH. This is because chronically altered expression of these molecules is often associated with diseases [49]. The concentrations of most of the cytokines/chemokines in our study participants were in the conventional normal ranges. Our previous studies showed that cytokine/chemokine cutoff concentrations for cognitive impairment in PLWH were in the conventional ranges [50]. Other studies have also shown that although plasma levels did not differ significantly between PLWH and healthy controls, within the PLWH group, pro-inflammatory cytokines were negatively correlated with cognitive function [51]. This implies that low-grade, chronic inflammation—levels that may still fall within the conventional “normal” clinical range—is sufficient to impair neuronal health, which may lead to cognitive impairment. A definitive interpretation of the data may be limited by the lack of clinically defined abnormalities in the plasma cytokine concentrations and their correspondence to brain volume. Therefore, future studies could be designed to characterize differences in systemic cytokine concentrations, normal versus abnormal, in relation to brain volume. This would clearly distinguish thresholds that have implications for brain pathology in PLWH.

Paired CSF and plasma cytokine/chemokine measures would provide a more proximal index of CNS involvement. However, like most cohort studies of PWH that have reported associations between brain integrity and peripheral cytokines, we did not have a sufficient number of CSF samples to examine cytokine levels in the central compartment. Therefore, we prioritized a clinically scalable, minimally invasive design (plasma + MRI). Paired CSF and plasma could have provided an opportunity to characterize the relationship of endogenous (CSF) cytokine levels and exogenous (peripheral) cytokine levels [52]. Lack of CSF measures in relation to brain volume is another limitation of this study. Therefore, future studies should analyse the impact of both peripheral cytokine levels and CSF levels on brain volume.

## 4. Materials and Methods

### 4.1. Study Participants

A retrospective cross-sectional study was conducted in 156 HIV-positive, Xhosa-speaking individuals recruited from primary care HIV clinics in Cape Town, South Africa, and that were being investigated for HANDs. Inclusion criteria to participate in the study required the following: (1) Age ranging from 18 and 45 years, with at least five years of formal education. This age range was selected to avoid age-related CNS abnormalities. (2) HIV serostatus, determined by ELISA and then confirmed by Western blot. (3) HIV-1 RNA for plasma viral load measured by the Abbott m2000sp and the Abbott m2000rt analysers (Abbott laboratories, Abbott Park, IL, USA). Exclusion criteria included the following: any major psychiatric condition that could significantly affect cognitive status; confounding neurological disorders, including multiple sclerosis and other CNS conditions; head injury with loss of consciousness for more than 30 min; clinical evidence of opportunistic CNS infections; and current substance abuse or alcohol abuse as defined by structured interviews [53]. Participants who were in the pre-treatment counselling phase were identified from clinic records. This group was chosen to evaluate the immune profile without the confounding effects of ART on these profiles. Interested participants completed a comprehensive consent form followed by clinical and demographic history. HIV-positive participants subsequently initiated treatment within 3 months of participating in the present study. This study was approved by the Health Research Ethics Committee (HREC) of Stellenbosch University, Ethics Reference # S17/02/035. 

### 4.2. Plasma Cytokine Quantification by Multiplex Assay

Cytokine concentration quantification in plasma samples was performed using a 27-plex Biorad Pro Human cytokine assay kit as described earlier [54]. Quantified cytokines included IL-1β, IL-2, IL-4, IL-5, IL-6, IL-7, IL-8, IL-9, IL-10, IL-12p70, IL-13, IL-15, IL-17, Eotaxin, basic FG, G-CSF, GM-CSF, IFN-γ, IP-10, MCP-1, MIP-1α, MIP-1β, PDGF-BB, RANTES, TNF-α, GM-CSF, and VEGF. The cytokine panel was chosen because it detects the most often researched and biologically relevant cytokines in a single cell, and it contains important cytokines involved in progression of HIV infection and associated comorbidities. Briefly, plasma samples were incubated with antibody-coupled beads. Complexes were washed, incubated with biotinylated detection antibody, and subsequently with streptavidin–phycoerythrin, prior to assessing cytokine concentrations. Standard curves were run together with samples by using standard cytokines provided in the kit. Plasma cytokine levels were determined using a multiplex array reader by Luminex^TM^ Instrumentation System (Bio-Plex Workstation by Bio-Rad, Hercules, CA, USA); plasma samples were run in duplicate, and cytokine concentrations were calculated as the average of two independent measures using Bioplex Manager Software, version 6.2.

### 4.3. Structural Neuroimaging and Volumetric Analysis

Acquisition of images and volumetric analysis of specific brain regions of interest (ROIs) were performed according to the methods described earlier [55]. Briefly high field MRI was used to acquire T1-weighted 3-dimensional magnetization-prepared rapid acquisition gradient echo (MPRAGE) images, which provide contrast for segmenting, grey matter, white matter, and CSF. The data obtained from MPRAGE were used for volumetric quantification of ROIs using the Freesurfer software suite (v5.1) (Martinos Center, Harvard University, Boston, MA, USA; http: //surfer.nmr.mgh.harvard.edu, accessed on 6 November 2017). Briefly, MP-RAGE scans were transformed into a template space with the skull stripped and the brain segmented into white matter, grey matter, and ventricles. The brain was further segmented into subcortical and cortical ROIs. Previous studies identify that these ROIs are impacted by HIV neuropathogenesis [56].

### 4.4. Statistical Analysis

Stata statistical package version 12.1 (StataCorp, College Station, Texas, USA, 2011) was used for all statistical analyses. Generalized linear modelling was used to determine the association between volume of brain regions of interest (ROI) and plasma cytokine levels. The Partial Least Squares Regression (PLS-R) approach, which uses multiple linear regression analysis to find the direction of maximum covariance between plasma cytokine levels and the volume of the ROIs, was used. Statistical significance was determined as a *p*-value < 0.05.

## 5. Conclusions

The observation that some pro-inflammatory cytokines were associated with lower cortical white matter and grey matter volume suggests that these cytokines are involved in neurodegenerative pathological processes in HIV infection. The associations between anti-inflammatory cytokines and growth factors with higher cortical white matter and grey matter volumes suggest that these cytokines have a neuroprotective or neurotrophic role. We suggest that those cytokines which were strongly associated with lower brain volume be investigated as potential biomarkers for HIV-associated neurodamage, whilst those associated with higher brain volume could be explored for immunotherapeutic approaches. While most participants were treatment-naïve, some had initiated ART within the past month of recruitment. As such, some of the early effects of first-line ART cannot be excluded. This study’s cross-sectional design also limits conclusions regarding causality. Longitudinal studies are, therefore, needed to observe changes in both cytokine levels and imaging data over time, so that the effect of cytokines on the brain can be determined. We also suggest that threshold analysis be performed to determine the cutoff values associated with each clinical outcome, as well as the effect of duration of HIV infection and CD4+ nadir on cytokine levels. For direct translation of cytokine–brain volume associations, future studies should include cognitive assessment data.

## Figures and Tables

**Table 1:** Study participants’ demographic and clinical characteristics.

Gender		Frequency (Freq) (n)		Percent (%)	Cumulative (%)	
Female		115		73.72	73.72	
Male		41		26.28	100.00	
Total		156		100.00		
Diagnosis						
Not impaired		98		63.24	63.24	
Impaired		58		36.76	100.00	
Total		156		100.00		
Treatment status						
No		112		82.96	82.96	
Yes		23		17.04	100.00	
Total		135		100.00		
Variable	Freq (n)	Mean	Std. Dev.	Median	Lower quartile (p25)	Upper quartile (p75)
Age	155	31.658	5.337	31.000	28	35
Viral load	107	124,750.27	292,383.8	21,515.000	3684	99,999
CD4 absolute	155	241.748	174.24	204.000	129	325

**Table 2:** Pairwise correlations between clinical parameters and brain volume (correlation coefficient, r, and associated *p*-values).

Variable	(1)	(2)	(3)	(4)	(5)	(6)	(7)	(8)	(9)
(1) Proviral load	1.000								
									
(2) THS ratio	−0.096	1.000							
	(0.264)								
(3) CD 45 Absolute	−0.046	−0.140	1.000						
	(0.593)	(0.103)							
(4) CD 348 absolute	−0.120	0.207 *	0.437 *	1.000					
	(0.163)	(0.016)	(0.000)						
(5) plasma viral load	0.364 *	−0.203 *	−0.137	−0.145	1.000				
	(0.000)	(0.018)	(0.110)	(0.091)					
(6) Subcortical grey matter	0.092	0.012	0.050	−0.045	0.105	1.000			
	(0.392)	(0.913)	(0.606)	(0.642)	(0.330)				
(7) Cortical white matter	−0.030	0.222 *	−0.020	0.014	−0.002	0.505 *	1.000		
	(0.784)	(0.038)	(0.836)	(0.886)	(0.985)	(0.000)			
(8) Total grey matter	0.083	0.127	0.006	−0.019	0.079	0.752 *	0.704 *	1.000	
	(0.443)	(0.238)	(0.947)	(0.846)	(0.466)	(0.000)	(0.000)		
(9) Whole_dti_mdm~n	−0.013	−0.254 *	−0.075	−0.133	0.184	−0.036	−0.294 *	−0.153	1.000
	(0.909)	(0.021)	(0.459)	(0.189)	(0.096)	(0.720)	(0.003)	(0.129)	

Comment: The number in brackets () is the *p*-value; * means statistically significant at the 5% level of significance.

**Table 3:** Adjusted correlations between cytokines levels and brain volume.

Cytokine	Coefficient	Std. Err.	z	*p* > |z|	95% Conf. Interval	
Cortical white matter volume						
IL-1β	0.0020477	0.0009955	2.06	0.040	0.0000966	0.0039988
IL-1α	4.44 × 10^−6^	1.67 × 10^−6^	2.66	0.008	1.17 × 10^−6^	7.70 × 10^−6^
IL-7	0.0010158	0.000482	2.11	0.035	0.000071	0.0019606
IL-9	1.42 × 10^−6^	6.33 × 10^−7^	2.24	0.025	1.80 × 10^−7^	2.66 × 10^−6^
MCP-1	−0.0009288	0.0003753	−2.47	0.013	−0.0016643	−0.0001933
PDGFBBB	3.57 × 10^−7^	1.42 × 10^−7^	2.52	0.012	7.89 × 10^−8^	6.36 × 10^−7^
RANTES	−1.52 × 10^−6^	4.82 × 10^−7^	−3.16	0.002	−2.47 × 10^−6^	−5.77 × 10^−7^
VEGF	1.57 × 10^−6^	4.93 × 10^−7^	3.19	0.001	6.07 × 10^−7^	2.54 × 10^−6^
Subcortical grey volume						
IL-1α	7.88 × 10^−6^	2.47 × 10^−6^	3.19	0.001	3.04 × 10^−6^	0.0000127
IL-6	−0.0021455	0.000831	−2.58	0.010	−0.0037743	−0.0005167
Total grey volume						
IL-1α	3.16 × 10^−6^	1.57 × 10^−6^	2.01	0.045	7.61 × 10^−8^	6.25 × 10^−6^
IL-6	−0.0020823	0.0005579	−3.73	0.000	−0.0031758	−0.0009888
IL-8	−0.0014577	0.0006186	−2.36	0.018	−0.0026701	−0.0002453
IL-10	−0.0035407	0.0013919	−2.54	0.011	−0.0062688	−0.0008126
IL-12p70	−0.0020964	0.0008961	−2.34	0.019	−0.0038527	−0.0003402
IL-15	−0.0001525	0.0000626	−2.44	0.015	−0.0002751	−0.0000298
GM-CSF	−0.003109	0.0010898	−2.85	0.004	−0.0052449	−0.0009732
GCSF	−0.0059812	0.0031477	−1.90	0.057	−0.0121505	0.0001881
MCP-1	−0.0024353	0.0008448	−2.88	0.004	−0.0040912	−0.0007795
RANTES	−2.54 × 10^−6^	1.04 × 10^−6^	−2.44	0.015	−4.57 × 10^−6^	−5.02 × 10^−7^
TNF-α	5.11 × 10^−6^	2.52 × 10^−6^	2.03	0.042	1.75 × 10^−7^	0.00001

## Data Availability

The original contributions presented in this study are included in the Supplementary Materials. Further inquiries can be directed to the corresponding author.

## Supplementary Materials

The following supporting information can be downloaded at: https://www.mdpi.com/article/10.3390/ijms262412000/s1.
