# Supplementary material for: Cortical White and Grey Matter Volume Differences Associated with Plasma Cytokine and Chemokine Levels in PLWH in Cape Town
Source: Int J Mol Sci. 2025 Dec 13;26(24):12000. doi: 10.3390/ijms262412000 (PMC12733071; doi:10.3390/ijms262412000)
Supplement: Supplementary file 1 [file ijms-26-12000-s001.zip › ijms-3874067-supplementary.pdf]

Supplementary Materials

S1:

**Table S1.** Standardized coefficients for cortical white matter volume in multiple GLM.

| Variable | Coefficient | Std. deviation | Lower bound (95%) | Upper bound (95%) | P value |
|---|---|---|---|---|---|
| IL1B | 0.008 | 0.023 | -0.038 | 0.054 | 0.147 |
| IL1RA | 0.026 | 0.026 | -0.026 | 0.078 | 0.627 |
| IL2 | -0.019 | 0.024 | -0.067 | 0.028 | 0.545 |
| IL4 | 0.005 | 0.027 | -0.049 | 0.059 | 0.815 |
| IL5 | -0.031 | 0.022 | -0.076 | 0.013 | 0.723 |
| IL6 | -0.043 | 0.019 | -0.082 | -0.005 | 0.281 |
| IL7 | 0.010 | 0.026 | -0.042 | 0.063 | 0.794 |
| IL8 | -0.014 | 0.024 | -0.061 | 0.034 | 0.694 |
| IL9 | 0.059 | 0.047 | -0.033 | 0.152 | 0.099 |
| IL10 | -0.024 | 0.015 | -0.055 | 0.006 | 0.240 |
| IL12P70 | -0.020 | 0.021 | -0.062 | 0.023 | 0.856 |
| IL13 | -0.001 | 0.023 | -0.047 | 0.044 | 0.955 |
| IL15 | -0.045 | 0.027 | -0.099 | 0.009 | 0.822 |
| IL17 | 0.032 | 0.034 | -0.036 | 0.100 | 0.693 |
| EOTAXIN | -0.019 | 0.023 | -0.066 | 0.027 | 0.429 |
| FGFBASIC | 0.013 | 0.031 | -0.048 | 0.073 | 0.648 |
| GCSF | 0.001 | 0.007 | -0.013 | 0.014 | 0.255 |
| GMCSF | -0.042 | 0.022 | -0.084 | 0.001 | 0.633 |
| IFNG | -0.008 | 0.027 | -0.062 | 0.046 | 0.898 |
| IP10 | -0.010 | 0.023 | -0.056 | 0.036 | 0.967 |
| MCP1 | -0.046 | 0.040 | -0.126 | 0.034 | 0.130 |
| MIP1A | -0.001 | 0.027 | -0.054 | 0.052 | 0.490 |
| PDGFBB | 0.034 | 0.045 | -0.055 | 0.123 | 0.688 |

| | | | | | |
|---|---|---|---|---|---|
| MIP1B* | 0.005 | 0.023 | -0.040 | 0.051 | 0.029 |
| RANTES | -0.044 | 0.047 | -0.137 | 0.049 | 0.694 |
| TNFA | 0.052 | 0.046 | -0.041 | 0.144 | 0.381 |
| VEGF | 0.048 | 0.044 | -0.038 | 0.135 | 0.155 |
| **Comment**: * mean statistically significant at 5% level of significance | | | | | |

**Table S2.** Standardized coefficients for subcortical grey matter volume in multiple GLM.

| Variable | Coefficient | Std. deviation | Lower bound (95%) | Upper bound (95%) | P value |
|---|---|---|---|---|---|
| IL1β* | 0.003 | 0.006 | -0.009 | 0.015 | 0.046 |
| IL1RA | 0.010 | 0.013 | -0.015 | 0.036 | 0.304 |
| IL2* | -0.008 | 0.014 | -0.035 | 0.020 | 0.012 |
| IL4 | 0.002 | 0.009 | -0.016 | 0.019 | 0.543 |
| IL5 | -0.012 | 0.018 | -0.049 | 0.024 | 0.216 |
| IL6* | -0.017 | 0.022 | -0.061 | 0.026 | 0.002 |
| IL7* | 0.004 | 0.007 | -0.010 | 0.018 | 0.014 |
| IL8* | -0.005 | 0.013 | -0.032 | 0.021 | 0.018 |
| IL9 | 0.023 | 0.024 | -0.024 | 0.071 | 0.419 |
| IL10 | -0.010 | 0.014 | -0.037 | 0.017 | 0.89 |
| IL12P70 | -0.008 | 0.015 | -0.038 | 0.022 | 0.81 |
| IL13 | -0.001 | 0.009 | -0.018 | 0.017 | 0.05 |
| IL15 | -0.018 | 0.021 | -0.060 | 0.024 | 0.821 |
| IL17 | 0.013 | 0.012 | -0.011 | 0.037 | 0.87 |
| EOTAXIN | -0.008 | 0.013 | -0.034 | 0.018 | 0.064 |
| FGFBASIC* | 0.005 | 0.008 | -0.010 | 0.020 | 0.039 |
| GCSF | 0.000 | 0.003 | -0.005 | 0.006 | 0.388 |
| GMCSF* | -0.016 | 0.020 | -0.057 | 0.024 | 0.030 |
| IFNG | -0.003 | 0.013 | -0.029 | 0.023 | 0.927 |

| | | | | | |
|---|---|---|---|---|---|
| IP10 | -0.004 | 0.010 | -0.024 | 0.016 | 0.072 |
| MCP1 | -0.018 | 0.019 | -0.056 | 0.020 | 0.481 |
| MIP1A | 0.000 | 0.010 | -0.020 | 0.019 | 0.249 |
| PDGFBB | 0.014 | 0.018 | -0.022 | 0.050 | 0.988 |
| MIP1B | 0.002 | 0.007 | -0.012 | 0.016 | 0.761 |
| RANTES | -0.017 | 0.021 | -0.059 | 0.024 | 0.655 |
| TNFA | 0.020 | 0.023 | -0.025 | 0.065 | 0.357 |
| VEGF | 0.019 | 0.017 | -0.016 | 0.054 | 0.705 |
| **Comment**: * mean statistically significant at 5% level of significance | | | | | |

**Table S3.** Standardized coefficients for total grey matter volume in multiple GLM.

| Variable | Coefficient | Std. deviation | Lower bound (95%) | Upper bound (95%) | P value |
|---|---|---|---|---|---|
| IL1β | 0.008 | 0.016 | -0.025 | 0.040 | 0.176 |
| IL1RA | 0.025 | 0.019 | -0.012 | 0.062 | 0.894 |
| IL2 | -0.018 | 0.029 | -0.077 | 0.040 | 0.605 |
| IL4 | 0.005 | 0.023 | -0.042 | 0.051 | 0.730 |
| IL5 | -0.030 | 0.030 | -0.091 | 0.031 | 0.444 |
| IL6* | -0.041 | 0.031 | -0.103 | 0.020 | 0.019 |
| IL7 | 0.010 | 0.018 | -0.025 | 0.045 | 0.144 |
| IL8* | -0.013 | 0.028 | -0.070 | 0.044 | 0.023 |
| IL9 | 0.057 | 0.025 | 0.008 | 0.106 | 0.826 |
| IL10 | -0.023 | 0.021 | -0.065 | 0.018 | 0.349 |
| IL12P70 | -0.019 | 0.027 | -0.073 | 0.035 | 0.778 |
| IL13 | -0.001 | 0.021 | -0.044 | 0.041 | 0.425 |
| IL15 | -0.043 | 0.031 | -0.104 | 0.017 | 0.592 |
| IL17 | 0.031 | 0.016 | -0.002 | 0.063 | 0.979 |
| EOTAXIN* | -0.019 | 0.025 | -0.068 | 0.031 | 0.035 |

| | | | | | |
|---|---|---|---|---|---|
| FGFBASIC | 0.012 | 0.021 | -0.030 | 0.054 | 0.651 |
| GCSF | 0.001 | 0.007 | -0.012 | 0.014 | 0.297 |
| GMCSF | -0.040 | 0.027 | -0.093 | 0.013 | 0.054 |
| IFNG | -0.008 | 0.029 | -0.066 | 0.051 | 0.626 |
| IP10 | -0.009 | 0.022 | -0.053 | 0.035 | 0.255 |
| MCP1 | -0.044 | 0.027 | -0.097 | 0.009 | 0.075 |
| MIP1A | -0.001 | 0.025 | -0.050 | 0.049 | 0.672 |
| PDGFBB | 0.033 | 0.030 | -0.028 | 0.093 | 0.532 |
| MIP1B | 0.005 | 0.019 | -0.033 | 0.044 | 0.216 |
| RANTES | -0.042 | 0.032 | -0.106 | 0.021 | 0.860 |
| TNFA | 0.050 | 0.026 | -0.003 | 0.102 | 0.347 |
| VEGF | 0.046 | 0.018 | 0.011 | 0.082 | 0.147 |
| **Comment**:  * mean statistically significant at 5% level of significance | | | | | |
